# Editorial: Understanding the Limitations of Current Influenza Vaccine Strategies and Future Development of More Efficacious Preventative and Therapeutic Interventions

**DOI:** 10.3389/fimmu.2019.02804

**Published:** 2019-11-29

**Authors:** Ralph A. Tripp, Abhijeet Bakre, John Stambas

**Affiliations:** ^1^Department of Infectious Disease, Animal Health Research Center, University of Georgia, Athens, GA, United States; ^2^School of Medicine, Deakin University, Geelong, VIC, Australia

**Keywords:** influenza virus, vaccine, efficacy, therapeutics, prevention

Influenza viruses pose a substantial threat to human health. Recent epidemics of seasonal influenza, poor vaccine efficacy, and the presence of zoonotic strains necessitate the need for disease intervention strategies to address transmission and infection. The development and implementation of novel intervention strategies to improve viral immunity and reduce the overall burden of disease are needed. Both the innate and adaptive immune systems contribute to influenza virus control and the virus itself employs various mechanisms to subvert effective innate and adaptive immune responses. As such, there is a need to address all aspects of viral immunology. This Research Topic highlights studies by experts in the field outlining current advances. A series of original research articles and a review article add to our understanding of limitations of current influenza vaccine and disease intervention strategies.

In a mini-review by Elbahesh et al. from the Guus F. Rimmelzwaan lab on “Response Modifiers: Tweaking the Immune Response Against Influenza A Virus,” the authors address the potential therapeutic use of different biological response modifiers (BRMs) as a way of modulating immune responses induced after IAV infections. BRMs are biologically active agents that include antibodies, small peptides, and/or other small molecules that can influence immunity. The authors discuss repurposing such agents and how this would allow for accelerated use against severe and potentially fatal IAV infections.

In an article by Enkirch et al. from Veronika von Messling's lab on “Identification and *in vivo* efficacy assessment of approved orally bioavailable human host protein-targeting drugs with broad anti-influenza A activity,” databases of known cellular targets of already licensed drugs were re-analyzed allowing for immediate deployment in the case of a pandemic, and the top licensed drugs characterized for anti-influenza activity in mice and ferrets. The anti-viral activity of these licensed drugs affected the viral life cycle and the compounds were orally bioavailable. Out of 15 candidate compounds, four were able to inhibit influenza virus infection 10- to 100-fold *in vitro* without toxicity. This study demonstrated the feasibility of a bioinformatics-driven rational approach for repurposing approved drugs against infectious diseases.

An article by Gautam et al. from the Hyung-Joo Kwon lab on how “Peritoneal cells mediate immune responses and cross-protection against influenza A virus” demonstrated that intraperitoneal inoculation with live influenza A virus confers protection against intranasal challenge in mice and ferrets. These results suggest that immunological responses in the peritoneal cavity are critical for protection against influenza A virus infection. The study shows that immunological responses in the peritoneal cavity in small animal models can induce effective protection against future virus infection. These findings are useful for the future development of candidate vaccines.

An article by Koutsakos et al. from Katherine Kedzierska's lab on “Downregulation of MHC class I expression by influenza A and B viruses” suggests that both IAV and influenza B virus (IBV) subtypes downregulate MHC I surface expression levels. These findings are highly relevant as manipulation of MHC class I presentation is thought to be a mechanism used by viruses to evade immune recognition. The findings provide insights into host-pathogen interactions and inform the design of novel vaccine strategies against influenza viruses.

Finally, an article by Hornick et al. from Kevin Legge's lab entitled “Kinetics and phenotype of the CD4 T cell response to influenza virus infections” describe the characterization of the full antigen-specific CD4+ T cell response using surrogate markers CD49d and CD11a to eliminate bias associated with the analysis of individual antigen specificity. The article highlights the importance of understanding the full diversity of the CD4+ T cell response associated with immunity following influenza virus infection and suggests that we consider all responses when formulating intervention strategies.

In summary, this collection of articles has identified several important approaches and identified strategic areas of interest to guide the development and optimization of future intervention strategies.

## Author Contributions

All authors listed have made a substantial, direct and intellectual contribution to the work, and approved it for publication.

### Conflict of Interest

The authors declare that the research was conducted in the absence of any commercial or financial relationships that could be construed as a potential conflict of interest.

